# Supplementary material for: Deletion of wfs1 Impairs Oligodendrocyte Precursor Cells Dorsal Distribution and Myelination Through the wfs1-hmgcs1 Axis in Zebrafish
Source: Biology (Basel). 2026 Aug 16;15(16):1402. doi: 10.3390/biology15161402 (PMC13510151; doi:10.3390/biology15161402)
Supplement: Supplementary file 1 [file biology-15-01402-s001.zip › File S1-Transcriptome sequencing data and differentially expressed genes (DEGs) in wfs1-- zebrafish at 5 dpf/2.KEGG/wfs1KOvsWT/UP/src/dre04530.html]

dre04530


- dre:792828

- Up regulated genes

792828\_1(7.34573383982123)

- dre:793158

- Up regulated genes

793158\_1(1.86160461223548)
- dre:556226

- Up regulated genes

556226\_1(6.36622858283894)

- dre:436718

- Up regulated genes

436718(1.34229427553561)

- dre:321070

- Up regulated genes

101882911(3.50047007681359)

- dre:677750

- Up regulated genes

677750(1.37913343503877)

- dre:100005566

- Up regulated genes

100005566(1.73719164936456)

- dre:559392

- Up regulated genes

559392\_1(5.40322756218621)

- dre:57934

- Up regulated genes

57934\_1(9.91304974129429)

- dre:57934

- Up regulated genes

57934\_1(9.91304974129429)

- dre:286739

- Up regulated genes

286739(1.28316470710399)

- dre:793143

- Up regulated genes

793143(6.32099639946357)

- dre:677750

- Up regulated genes

677750(1.37913343503877)

- dre:793952

- Up regulated genes

793952\_1(1.28875020528538)

- dre:436718

- Up regulated genes

436718(1.34229427553561)

- dre:793158

- Up regulated genes

793158\_1(1.86160461223548)
- dre:556226

- Up regulated genes

556226\_1(6.36622858283894)

- dre:431777

- Up regulated genes

431777(1.35055689210441)

- dre:57934

- Up regulated genes

57934\_1(9.91304974129429)

- dre:436718

- Up regulated genes

436718(1.34229427553561)

- dre:797651

- Up regulated genes

797651\_1(1.84553757065454)
- dre:100005566

- Up regulated genes

100005566(1.73719164936456)

- dre:797651

- Up regulated genes

797651\_1(1.84553757065454)
- dre:100005566

- Up regulated genes

100005566(1.73719164936456)

- dre:793143

- Up regulated genes

793143(6.32099639946357)

- dre:793143

- Up regulated genes

793143(6.32099639946357)

- dre:793143

- Up regulated genes

793143(6.32099639946357)

- dre:793143

- Up regulated genes

793143(6.32099639946357)

- dre:792828

- Up regulated genes

792828\_1(7.34573383982123)

- dre:57934

- Up regulated genes

57934\_1(9.91304974129429)

- dre:100005566

- Up regulated genes

100005566(1.73719164936456)

- dre:100005566

- Up regulated genes

100005566(1.73719164936456)

- dre:100005566

- Up regulated genes

100005566(1.73719164936456)

- dre:337226

- Up regulated genes

337226\_1(4.74603512488049)
- dre:798632

- Up regulated genes

798632(1.33935841885554)

- dre:321070

- Up regulated genes

101882911(3.50047007681359)

- dre:677750

- Up regulated genes

677750(1.37913343503877)

- dre:793158

- Up regulated genes

793158\_1(1.86160461223548)
- dre:556226

- Up regulated genes

556226\_1(6.36622858283894)

- dre:559392

- Up regulated genes

559392\_1(5.40322756218621)

Close
